# Hyperreflective foci on optical coherence tomography associate with treatment outcome for anti-VEGF in patients with diabetic macular edema

**DOI:** 10.1371/journal.pone.0206482

**Published:** 2018-10-31

**Authors:** Vivian Schreur, Lebriz Altay, Freekje van Asten, Joannes M. M. Groenewoud, Sascha Fauser, B. Jeroen Klevering, Carel B. Hoyng, Eiko K. de Jong

**Affiliations:** 1 Department of Ophthalmology, Donders Institute for Brain, Cognition and Behaviour, Radboud University Medical Center, Nijmegen, The Netherlands; 2 Department of Ophthalmology, University Hospital of Cologne, Cologne, Germany; 3 Department of Health Evidence, Radboud University Medical Center, Nijmegen, The Netherlands; Justus Liebig Universitat Giessen, GERMANY

## Abstract

**Purpose:**

To investigate the relationship between baseline number of hyperreflective foci (HF) on spectral domain optical coherence tomography (SD-OCT) in patients with diabetic macular edema (DME), as well as the dynamics of HF during treatment with anti-vascular endothelial growth factor (VEGF), and treatment response.

**Methods:**

We evaluated patients diagnosed with DME scheduled for treatment with intravitreal bevacizumab. Eyes were classified as adequate or insufficient treatment responders based on logMAR visual acuity improvement and central retinal thickness (CRT) decrease after three consecutive injections. Associations between number of HF at baseline and treatment response, the change in HF over the course of treatment, and the distribution of HF within the retinal layers were evaluated.

**Results:**

In 54 eyes of 41 patients, mean number of HF and CRT decreased after intravitreal treatment with bevacizumab (p = 0.002 and p<0.001 respectively). Decrease in CRT after 3 months was independently associated with a higher number of HF at baseline (estimated effect -2.61, 95% CI [-4.42–-0.31], p = 0.006). Eyes with adequate treatment response presented with more HF at baseline (OR 1.106, 95% CI [1.012–1.210], p = 0.030) than eyes with insufficient treatment response. Most HF were located within the inner retinal layers, and decrease of HF was mostly due to a decrease of inner retinal HF.

**Conclusions:**

In patients with DME treated with anti-VEGF, higher baseline numbers of HF have predictive value for treatment response in terms of visual acuity improvement and CRT decrease after 3 months. In addition, HF were responsive to anti-VEGF therapy.

## Introduction

Diabetic macular edema (DME) is a sight threatening complication of diabetes mellitus (DM) and one of the most frequent causes of vision loss.[1] Because vascular endothelial growth factor (VEGF) plays a central role in the development of centre-involved DME, anti-VEGF agents have been implemented as the treatment of choice for this condition. However, not all patients respond equally well to the initiated treatment, in which case patients are redirected to treatment with an alternative anti-VEGF agent or long acting corticosteroids.[2] Currently, we are not able to choose the best treatment option for an individual patient a priori, because information on baseline characteristics that associate with treatment outcomes is lacking. Any delay in finding the most effective personalized treatment strategy may result in irreversible visual impairment and also increases the costs of health care.[3]

Although visual function is the most relevant outcome measure, it is a subjective measure of treatment response, and can be influenced by for example fluctuations in glucose levels or the presence of other ocular disorders. Conversely, anatomical measurements such as central retinal thickness (CRT) on spectral domain optical coherence tomography (SD-OCT) are a more objective and reliable outcome measure for treatment response. The diabetic retinopathy clinical research network (DRCR.net) employs a combination of both outcome measures and defined insufficient treatment response as a CRT decrease of ≤10%, or a gain of ≤5 letters on the Early Treatment of Diabetic Retinopathy Study (ETDRS) chart, the equivalent of 0.1 logMAR.[4, 5]

Hyperreflective foci (HF) are well-circumscribed dots that can be visualized on SD-OCT in all retinal layers. They were first described in patients with DM by Bolz et al. and have since been associated with the presence of DME, as well as with non-proliferative stages of diabetic retinopathy.[6, 7] Hypotheses about their etiology diverge: some authors suggested they could be lipid extravasations acting as subclinical hard exudates.[6, 8, 9] Others have argued that HF are migrating RPE cells since the reflectivity of HF corresponds with that of the RPE,[10] or that they might be degenerated photoreceptor cells.[11] Another theory is that HF are aggregates of cells involved in retinal inflammatory response, such as activated microglia.[12]

The purpose of this study was to investigate the association between baseline number of HF and treatment response to anti-VEGF in terms of visual acuity (VA) improvement and CRT decrease. We also studied the location of HF in the neuroretina and the behavior of HF during anti-VEGF treatment.

## Material and methods

### Population

We reviewed the medical files of DM type 2 patients with DME who were treated with intravitreal injections of bevacizumab (1.25 mg) at the department of Ophthalmology of the Radboud University Medical Center between November 2010 and May 2013. We restricted inclusion to treatment naive patients who received a complete loading dose of three consecutive injections with a 4–6 weeks interval, and of whom baseline and 3 month follow up data were available. Other exclusion criteria were: laser treatment or intraocular surgery within 12 weeks prior to the first injection, active proliferative diabetic retinopathy, vitreous hemorrhage or tractional retinal detachment at baseline visit, and presence of other retinal vascular diseases. This study adhered to the tenets of Helsinki. The Research Ethics Committee of the Radboud university medical center Nijmegen approved this study and waived the requirement for informed consent (2018–4424). All data was fully anonymized before it was accessed by the investigators.

### Data collection

The following clinical characteristics were assessed: gender, age, duration of DME, and DR staging according to International Clinical Diabetic Retinopathy Severity Scale.[13] At baseline and 3 month visits, Snellen VA was measured and converted to the logarithm of the maximum angle of resolution (logMAR). We used SD-OCT-scans (Spectralis HRA+OCT, Heidelberg Engineering, Heidelberg, Germany) to automatically measure CRT as the average thickness in an area of 1000 μm diameter surrounding the foveal centre. Adequate response was defined as a CRT decrease of >10% and a VA improvement of >0.1 logMAR.[4, 5]

### Image grading

**Fig 1:**
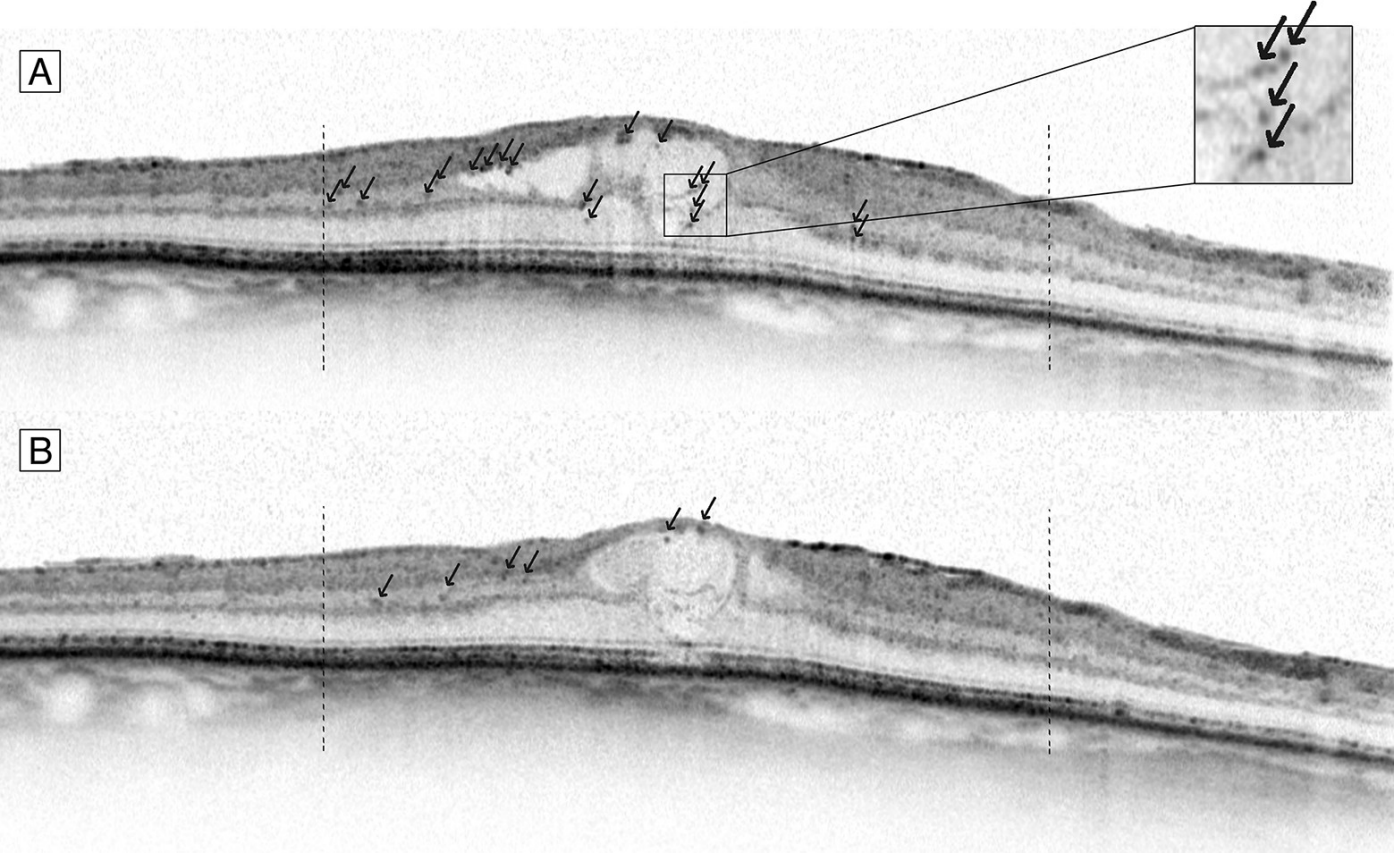
Hyperreflective foci on SD-OCT before and after treatment with anti-VEGF. Foveal centered spectral domain optical coherence tomography (SD-OCT) B-scan image of a patient with DME before (A) and after (B) 3 injections with anti-VEGF. Black arrows indicate hyperreflective foci, within 3000 μm of the fovea (dashed bars).

We selected baseline and 3 months B-scans centered on the fovea and evaluated these scans for the presence of HF within the central 3000 μm around the fovea (Fig 1). HF were defined as small, round or oval-shaped, well-circumscribed dense particles with higher reflectivity than the background. The size could not exceed 100 μm, as we hypothesized this to be clumps that can be visualized as hard exudates on fundus photographs. The total number of HF in each scan was counted, as well as the number of foci within the inner (inner nuclear layer) and outer (outer plexiform layer to outer border of the external limiting membrane) retinal layers. The nerve fiber layer through the inner plexiform layer, and the photoreceptor layer and the retinal pigment epithelium (RPE) were excluded from analysis, because the naturally high reflectivity of these layers impedes the evaluation of HF. Additionally, the number of HF with a higher reflectivity than the RPE were counted. Grading was performed by two experienced independent graders (LA, VS), masked to all clinical information. In case of disagreement in counted number of HF between the two graders exceeded 20%, differences were resolved through discussion. For all other observations, the average of both graders was used for analysis. In addition, the observers graded the central B-scans for the presence of cysts, subretinal fluid, foveal disruption of the external limiting membrane and the photoreceptor layer, and the presence of disorganization of the retinal inner layers (DRIL).

### Statistical analysis

An intraclass correlation coefficient for the number of HF was calculated to assess interrater agreement. To account for the relationship between both eyes, univariable linear mixed model analyses were used to assess the difference in CRT, VA and number of HF before and after treatment, and to evaluate the association between baseline number of HF and (change in) CRT and VA. To correct for potential confounders, multivariable linear mixed model analyses were used, applying stepwise backward deletion of non-significant confounders with an influence of <10% on the estimate of HF at baseline. These analyses were conducted using SPSS version 22 (SPSS, Chicago, IL, USA). Mixed effect logistic regression analysis was used to assess associations between baseline number and change of HF with adequate or insufficient treatment response, expressed as an odds ratio (OR) with 95% confidence interval (CI). These analyses were performed in SAS Statistical Analysis Software 9.2 (SAS Institute, Cary, NC, USA). P-values <0.05 were considered statistically significant.

## Results

**Fig 2:**
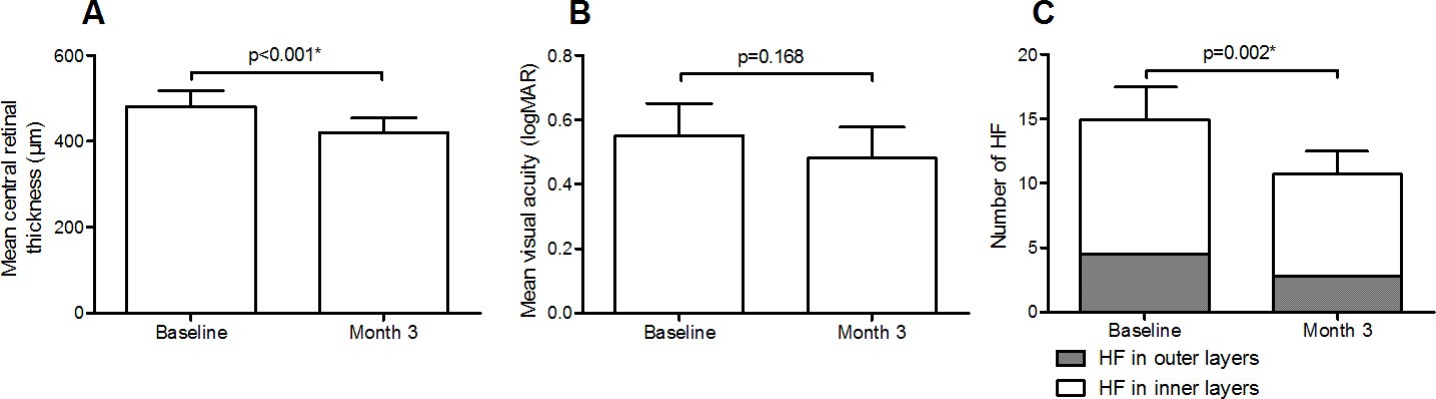
Differences between before and after treatment with anti-VEGF. Mean changes in central retinal thickness (A), visual acuity (B) and number of hyperreflective foci (C) at baseline and after 3 injections with anti-VEGF. The bars represent mean ± 95% confidence interval. *P<0.05.

Fifty-four eyes of 41 subjects were evaluated over the course of 3 months of treatment with bevacizumab. Patient characteristics at baseline are described in Table 1. Mean CRT at baseline was 482 ± 128 μm, and decreased significantly after treatment with bevacizumab to 419 ± 127 μm (p<0.001, Fig 2A). LogMAR VA was 0.54 ± 0.36 (Snellen 20/69) at baseline and improved to 0.48 ± 0.35 (Snellen 20/60) at the 3 month visit, although this difference did not reach statistical significance (p = 0.168, Fig 2B). For HF measurements, the intraclass correlation coefficient was 0.85 [0.79–0.89]. Mean number of HF at baseline was 14.8 ± 9.7, and decreased to 10.7 ± 6.5 (p = 0.002, Fig 2C).

**Table 1:** Baseline characteristics.

Variable (n = 54 eyes of 41 subjects)	
Gender, n (%)	
Male	22 (54%)
Female	19 (46%)
Age, mean (SD), years	67 (12)
Duration DME per eye, median (IQR), months	8 (2–21)
DR stage per eye, n (%)	
Mild	9 (17%)
Moderate	41 (76%)
Severe	4 (7%)
Presence of cysts per eye, n. (%)	51 (94%)
Presence of subretinal fluid per eye, n (%)	13 (24%)
Foveal disruption of ELM per eye, n (%)	20 (37%)
Foveal disruption of PR layer per eye, n (%)	21 (39%)

n = number; SD = standard deviation, DM = diabetes mellitus; DME = diabetic macular edema; IQR = interquartile range; DR = diabetic retinopathy; ELM = external limiting membrane; PR = photoreceptor

HF were scattered throughout all retinal layers. The mean number of HF in the inner retinal layers at baseline was 10.4 ± 7.3 versus 7.9 ± 5.0 after treatment (p = 0.010, Fig 2C). In the outer layers the mean number of HF at baseline was 4.5 ± 4.9 compared to 2.8 ± 3.0 after treatment (p = 0.013, Fig 2C). The percentage of HF with a reflectivity higher than the RPE was higher in the outer retinal layers (64% at baseline and 68% at 3 months) than in the inner retinal layers (36% at baseline and 25% at 3 months).

The number of HF at baseline was independently associated with a decrease in CRT after 3 months (p = 0.006, Table 2). An estimate of -2.61 was found for this association, implicating that for every HF counted at baseline, CRT declines with 2.61 μm after treatment. There was no relation between the number of HF at baseline and baseline CRT (Table 2). When we investigated the relationship between HF number at baseline and VA outcome measures, we found that baseline VA was 0.008 points worse for every counted HF (p = 0.047). No effect of baseline number of HF and change in VA after 3 months was found (Table 2).

**Table 2:** Linear mixed model analysis of the effect of baseline number of HF on baseline values of VA and CRT, and VA and CRT changes.

	Univariable			Multivariable		
	Estimate	95% CI	P	Estimate	95% CI	P
CRT baseline, μm	-1.89	-5.71–1.94	0.325	-3.54	[-7.44–0.366]	0.074
CRT change, μm	-2.47	-4.64 –-0.31	0.026*	-2.61	[-4.42–0.79]	0.006*
VA baseline, logMAR	0.013	0.004–0.023	0.008*	0.008	[0.001–0.016]	0.047*
VA change, logMAR	-0.002	-0.009–0.004	0.473	-0.002	[-0.006–0.009]	0.661

Multivariable analyses were corrected for gender, age, duration of DME, DR stage, presence of cysts, presence of subretinal fluid, disruption of the external limiting membrane, disruption of the photoreceptor layer and the presence of disorganization of the retinal inner layers. Analyses of CRT and VA changes were also corrected for both CRT baseline and VA baseline. Analysis of CRT baseline was also corrected for VA baseline, and analysis of VA baseline was also corrected for CRT baseline.

CI = confidence interval; HF = hyperreflective foci; CRT = central retinal thickness

*P<0.05.

**Fig 3:**
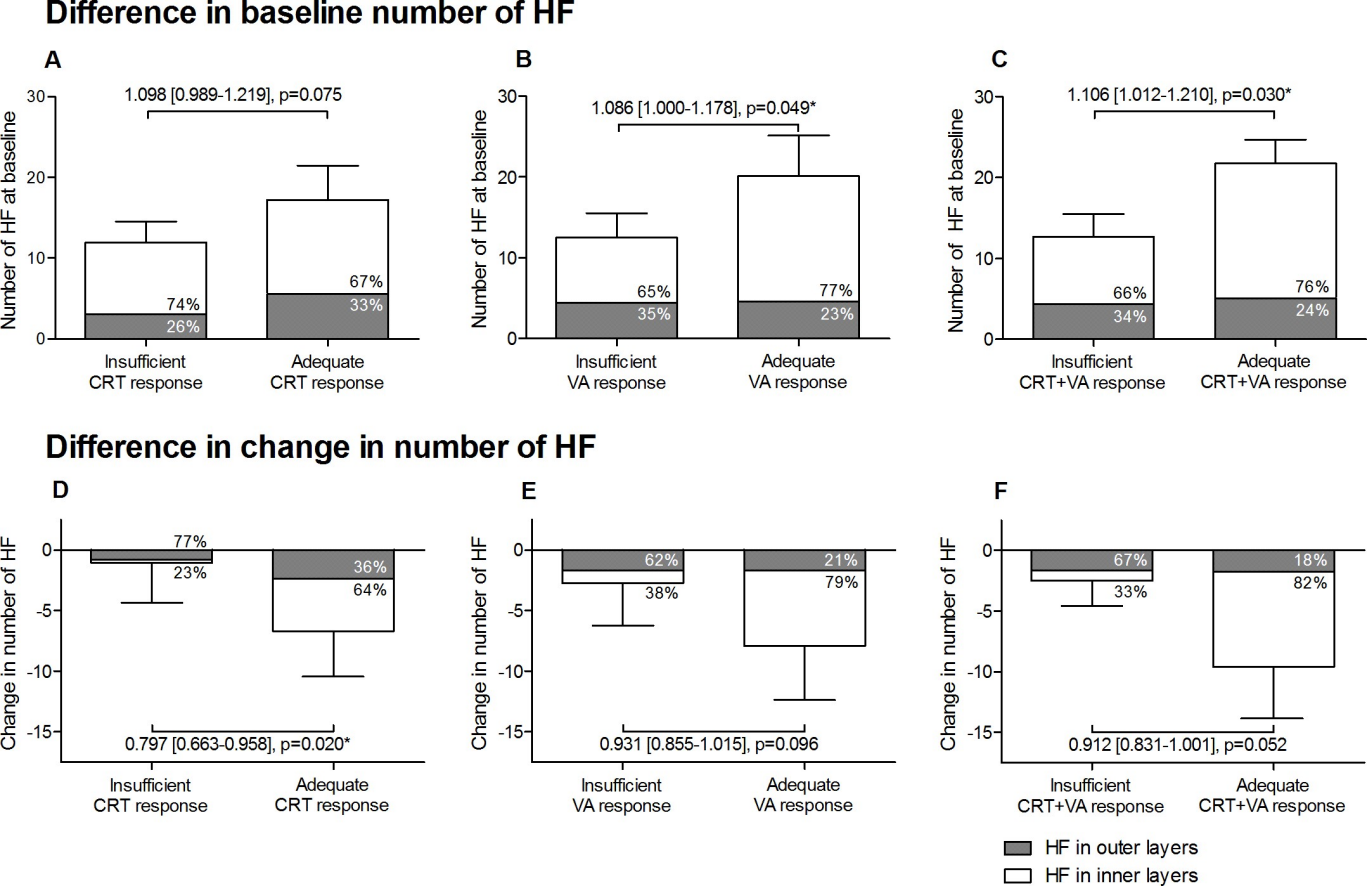
Difference in number of HF at baseline, and change in HF between eyes with adequate response versus insufficient response. Mean number of HF at baseline in groups based on insufficient and adequate CRT response (A); insufficient and adequate VA response (B); and insufficient and adequate combined CRT + VA response (C). Mean change in number of HF in groups based on insufficient and adequate CRT response (D); insufficient and adequate VA response (E); and insufficient and adequate combined CRT + VA response (F). The distribution of HF in the inner and outer retinal layers is displayed as a percentage. The values represent the odds ratio with corresponding 95% confidence interval and p-value. The odds ratio of adequate response is 1.106, which can be interpreted as an increase, or chance, for adequate response by 10.6% for every HF at baseline; when the number of HF at baseline increases by 10, the chance for adequate response increases exponentially by 1.106^10^ = 2.74 or 274%. The bars represent mean ± 95% confidence interval and are based on descriptive statistics. *P<0.05.

Next, we stratified the cohort into two groups with either adequate or insufficient treatment response. A total of 30 eyes (56%) showed a CRT decrease of ≥10%, while 16 eyes (30%) showed an improvement in VA of ≥0.1 logMAR. Subsequently, 13 eyes (24%) met both CRT and VA criteria for adequate treatment response, classifying 41 eyes (76%) as insufficient responders. Eyes that were classified as adequate responders presented with higher numbers of HF at baseline than eyes with insufficient CRT and VA response (21.6±9.5 vs. 12.7±8.8, OR 1.106, 95% CI [1.012–1.210], p = 0.030, Fig 3A–3C). In eyes showing adequate CRT and VA response, 76% of HF were observed in the inner retinal layers, while this amounted to 66% in eyes with insufficient response (Fig 3C).

We did not observe a significant difference in HF change between eyes with an adequate CRT and VA response and eyes with insufficient CRT and VA response (-9.5±7.4 vs. -2.6±8.8, OR 0.912, 95% CI [0.831–1.001], p = 0.052, Fig 3D–3F). In eyes showing an adequate response, the decrease in HF was mostly seen in the inner retinal layers, whereas in insufficient responders this decrease was more prominent in the outer retinal layers (Fig 3F).

## Discussion

Higher numbers of HF at baseline were associated with adequate treatment response to anti-VEGF, in terms of CRT decrease and VA improvement. The relationship between baseline number of HF and CRT decrease was also apparent in linear mixed model analysis, showing that the effect was independent from potential confounders. In addition, HF were responsive to treatment with anti-VEGF, and were more often detected in the inner retinal layers than in the outer retinal layers. We also observed that a high number of HF at baseline was associated with poorer VA prior to treatment with anti-VEGF.

The observation that higher numbers of HF at baseline are associated with adequate treatment response could be of importance for clinical practice. For example, when poor response to anti-VEGF can be predicted based on baseline biomarkers, the decision to start with an alternative treatment can be made beforehand, thereby preserving visual performance. Ideally, these findings should be integrated in a prediction model that can reliably predict treatment response, including all other potential predictive factors, such as patient characteristics, environmental factors, genotype variables and other imaging biomarkers to be further investigated.

To the best of our knowledge, a relationship between high baseline number of HF and both adequate morphological and functional treatment outcomes in DME has not been established before, although it has been studied in other cohorts. An association between higher numbers of HF and final visual acuity at a mean of 7 months after treatment with bevacizumab in 33 eyes with DME accompanied by serous detachment was reported by Kang et al.[14] Contrarily, Hwang et al. recently reported fewer numbers of HF to be associated with good CRT response after 3 months of bevacizumab treatment. The reason for this disparity is unclear, although there are substantial differences in study design.[15] Hwang et al. studied particles with equal or higher reflectivity than the RPE, while we used a reflectivity higher than the surrounding tissue for a definition, which in our experience results in a more reliable detection of HF. Furthermore, proliferative diabetic retinopathy is associated with an increased inflammatory response and exuberant activation of microglia, and we therefore hypothesize that neovascularization could influence the number of HF.[16] For this reason, we excluded patients with active neovascularization, while DR staging was not reported by Hwang et al. Vujosevic et al., who also studied the relationship between baseline HF and treatment response, did not find a association between baseline number of HF and change in CRT or VA after treatment with ranibizumab in respectively 20 and 26 eyes with DME, which might be due to small sample size.[17, 18] Moreover, different criteria for treatment response were used by all study groups: Hwang et al. chose for a treatment response definition of CRT <300 μm or a reduction by more than 50 μm, while Kang et al. and Vujosevic et al. defined treatment outcome by the continuous variables VA improvement and CRT reduction, respectively.[14, 15, 17, 18] We chose a definition of CRT decrease of >10% and a VA improvement of >0.1 logMAR, as proposed by the Diabetic Retinopathy Clinical Research Network, as a CRT reduction expressed as a percentage accounts for differences in baseline CRT, and the combination with VA improvement provides valuable information on visual function.[4, 5]

The association between higher baseline number of HF and poorer baseline VA is in line with previous studies,[11, 19] and could be a relevant observation for clinical practice. Often, vision loss is not the first manifestation of DME. Therefore, large-scaled screening programs are set up for patients with DM to detect early changes related to leakage of fluid into the retina, such as microaneurysms and hard exudates.[20] It has been suggested that HF are the missing link between the breakdown of the inner blood–retina barrier and the osmotic swelling of retinal layers.[8] HF, as an earlier representation of microvascular damage, may be important in the risk assessment for progression of DME and vision loss.

Various different hypotheses have emerged about the origin of HF that might in fact coexist. HF could be precursors of hard exudates,[6, 8, 9] migrating RPE cells,[10] degenerated photoreceptor cells,[11, 19] or aggregations of activated immune cells, such as microglia.[12] Microglia are highly ramified phagocytic cells in the retina and are required for neuronal homeostasis and immune defense. VEGF has been demonstrated to induce microglial activation,[21] engaging a feedback loop in which immune cells in the retina simultaneously express and release VEGF.[22] Our work shows that HF are responsive to anti-VEGF and reside predominantly in the inner retinal layers, which is in accordance with microglial cell behavior.[16, 23] Moreover, the decrease in HF after 3 months of bevacizumab was most prominent in the inner retinal layers, which supports the hypothesis that HF in these layers correspond to activated microglia, and that HF in the outer retinal layers may represent a different entity. It has also been hypothesized that HF are precursors of hard exudates, given the equal reflectivity on SD-OCT. Hard exudates are commonly located in the outer plexiform layer, and unlike microglial cells, they are not subject to rapid regression after only 3 months of treatment, although these dynamics are unknown for precursors of hard exudates.[12, 24, 25] In the outer retinal layers, we found that most HF had a reflectivity higher than the RPE, in contrast to the HF in the inner retinal layers. The unresponsiveness to therapy and the hyperreflective appearance suggests that at least some of the HF in the outer layers are precursors of hard exudates or migrating RPE cells, as was proposed earlier.[10] Further research is warranted to study the etiology and clinical value of HF, by means of histological and epidemiological studies.

Overall, there was a significant decrease in CRT, but no significant improvement in VA after 3 injections of bevacizumab in this cohort. Earlier studies demonstrated that treatment outcomes in real life are often inferior to the results of controlled clinical trials.[26, 27] Moreover, the follow up duration in this study was relatively short, and it is known from large clinical trials that VA can improve even after the loading dose of 3 injections before stabilizing.[5] Most large clinical trials include both patients with type 1 or type 2 diabetes. In our study, we only included patients with type 2 diabetes, which may explain why these patients are slightly older than in most clinical trials. However, to the best of our knowledge, there is no evidence from previous studies that age or type of diabetes are associated with worse treatment outcomes.[28]

The use of a well-designed, detailed grading protocol was one of the major strengths of this study, evidenced by the good interrater agreement. This protocol enables replication of our findings and validation in other cohorts. Another strength was the well-defined cohort: we used strict inclusion criteria to limit the analysis to eyes with a completed loading phase and follow-up. Sample size and the short duration of follow-up can be considered limitations of our study, although it has been shown that VA response at 3 months is highly predictive for long term outcomes of anti-VEGF therapy.[29] Another limitation of this study is the grading of the central horizontal SD-OCT B-scan only, thus we may not be able to generalize our findings to peripheral parts of the macula. The process of HF grading is labor-intensive, and a computer assisted approach could facilitate fast and replicable grading. Furthermore, the development of software-based grading is inevitable when translating HF grading from research to clinical practice, in order to implement feasible evaluation of HF for the clinician.

In conclusion, our results suggest that in patients with DME, baseline number of HF are correlated with visual acuity as well as anti-VEGF treatment response in terms of CRT reduction and VA improvement. Replication of our findings in larger cohorts is needed to assess the validity of our study. Additionally, the relation between HF and other anti-VEGF agents and corticosteroids needs to be further explored. This knowledge may contribute to the development of proper risk assessment, and therefore enable personalized decision making and prevention of overtreatment.

## Supporting information

S1 Dataset Dataset used for statistical analysis. (SAV)
